# Supplementary material for: Fold or flop: quality assessment of AlphaFold predictions on whole proteomes
Source: Bioinform Adv. 2026 Jul 8;6(1):vbag190. doi: 10.1093/bioadv/vbag190 (PMC13426001; doi:10.1093/bioadv/vbag190)
Supplement: vbag190_Supplementary_Data [file vbag190_supplementary_data.pdf]

1 Supporting information

1.1 Annex

1.1.1 Inclusion radius

There is no consensus in the literature on a C_α–C_α distance threshold for defining neighboring residues, which is typically chosen in the range 8–14 Å depending on the application. We calculate the two-way trustworthiness between k-neighborhoods ($k = \{10, 20, 50, 100\}$) in the PCA and arity map spaces and find values in the range $[0.95, 0.97]$ for any radius between 6 Å (capturing only secondary structure contacts) and 14 Å (capturing the second residue shell). As a compromise between informativeness and computational cost, we fix the neighborhood distance threshold at 10 Å; all quartile values and references to the arity map hereafter use this threshold.

1.1.2 Wasserstein PCA and arity map embedding

q_1	q_2	Spearman coeff.
10	60	0.919
10	70	0.944
10	80	0.943
10	90	0.905
20	60	0.930
20	70	0.960
20	80	0.970
20	90	0.948
30	60	0.937
30	70	0.967
30	80	**0.980**
30	90	0.967
40	60	0.936
40	70	0.965
40	80	0.978
40	90	0.970

Table 1: **Grid search over the percent values of the arity map quantiles.** We calculate the Spearman correlation coefficient between the 2-dimensional PCA of $\mathcal{W}_2$ distances and the arity map, a 2D-histogram defined over two quantiles corresponding to the i and j percentages. The best correlation coefficient between the two 2D dimensionality reductions corresponds to the (30, 80) percent pair, thus selecting quantiles very close to the first and third quartile.

1.1.3 Arity distribution for typical folds

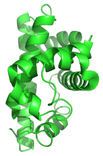

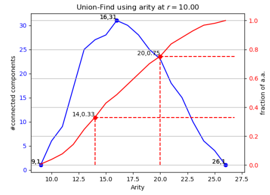

Fold α

Globin, 154 a.a.

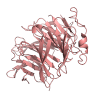

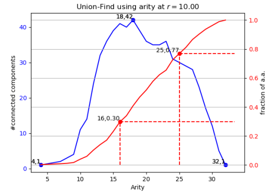

Fold β

Propeller, 350 a.a.

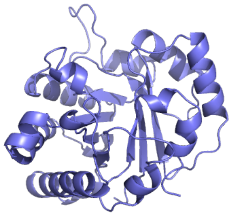

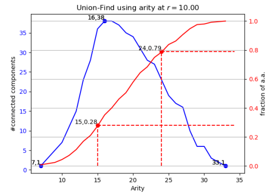

Fold α/β

TIM barrel, 247 a.a.

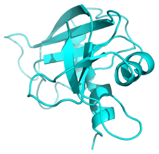

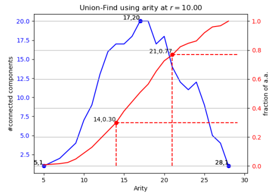

Fold $\alpha + \beta$

Ribonuclease, 124 a.a.

Figure 1: **Arity distribution and arity signature for prototypical folds.** Fold α: 101m-globin-alpha; 154 amino acids. Quantile-arity signature [(0.25, 14), (0.5, 17), (0.75, 20)] Fold β: 1erj-propeller-beta; 350 amino acids. Quantile-arity signature [(0.25, 16), (0.5, 20), (0.75, 25)] Fold α/β: 8tim-TIMbarrel-alphaSlashBeta; 247 amino acids. Quantile-arity signature [(0.25, 15), (0.5, 19), (0.75, 24)] Fold $\alpha + \beta$: 1a5p-ribonuclease-alphaPlusBeta; 124 amino acids. Quantile-arity signature [(0.25, 14), (0.5, 17), (0.75, 21)]

1.2 pLDDT statistics per organism

For the reference, this section provides simple analysis on pLDDT values.

Let us recall the following intervals for pLDDT values: very high: $0.9 \leq$ pLDDT; high: $0.7 \leq$ pLDDT < 0.9; low: $0.5 \leq$ pLDDT < 0.7; very low pLDDT < 0.5.

While the distribution of pLDDT values has been studied in selected cases, in particular for H. Sapiens in the context of conditionally folding proteins, we perform instead a systematic study on all genomes. We process all predictions (PDB files from `AlphaFold-DB`) of a given organism, collecting, per prediction, all/the median/the mean pLDDT value(s). These values yield three distributions, namely that for (i) all pLDDT values of all a.a. of all proteins, (ii) all median pLDDT values, and (iii) and all mean pLDDT values (Table 2).

Per genome statistics show that the fraction of a.a. with pLDDT below 0.7 lies in the range 0.078-0.408 and is rather consistent for model organisms (Table 2). However, the inspection of other organisms shows major discrepancies, with a value as low as 0.086 for *P. aeruginosa* and as high as 0.584 for *P. falciparum* (Table 2, Table 4, Table 5)

Selected illustrations are provided below.

genome / pLDDT	50	70	90
AThaliana/all	0.204	0.315	0.550
CAlbicans/all	0.213	0.312	0.570
CElegans/all	0.202	0.323	0.597
DDiscoideum/all	**0.288**	0.423	**0.681**
DMelanogaster/all	0.281	0.387	0.623
DRerio/all	0.246	0.343	0.593
EColi/all	**0.029**	**0.078**	0.275
GMax/all	0.217	0.338	0.577
HSapiens/all	0.284	0.382	0.666
MJannaschii/all	0.035	0.084	**0.264**
MMusculus/all	0.255	0.352	0.597
OryzaSativa/all	0.257	**0.408**	0.623
RattusNorvegicus/all	0.253	0.351	0.596
SCerevisiae/all	0.213	0.314	0.582
SPombe/all	0.187	0.289	0.558
ZeaMays/all	0.260	0.394	0.635
SAureus/all	0.045	0.096	0.295
HPylori/all	0.053	0.123	0.347
MTuberculosis/all	0.069	0.133	0.322
Aeruginosa/all	**0.036**	**0.086**	**0.278**
PFalciparum/all	**0.460**	**0.584**	**0.804**

Table 2: **Cumulated distribution function for pLDDT values of all a.a. of all proteins in a genome.** Top: model genomes. Bottom: five *global health* proteomes. Min and max values per column stressed in bold.

1.2.1 HSapiens

As reported in Figure 2 and Table 3:

- Worst cases, top 3: ['File HSapiens/AF-Q96MU5-F1-model_v4.pdb has 243 a.a.; pLDDT min/med/max: 19.88 25.35 38.27', 'File HSapiens/AF-Q6ZR03-F1-model_v4.pdb has 302 a.a.; pLDDT min/med/max: 21.04 26.27 46.20', 'File HSapiens/AF-Q96M85-F1-model_v4.pdb has 177 a.a.; pLDDT min/med/max: 20.02 26.32 42.34']

- Median cases, 3 of them: ['File HSapiens/AF-P56159-F1-model_v4.pdb has 465 a.a.; pLDDT min/med/max: 25.40 86.54 98.58', 'File HSapiens/AF-Q96N96-F1-model_v4.pdb has 652 a.a.; pLDDT min/med/max: 24.37 86.54 98.41', 'File HSapiens/AF-Q9H009-F1-model_v4.pdb has 215 a.a.; pLDDT min/med/max: 36.28 86.54 95.84']

- Top cases, top 3: ['File HSapiens/AF-Q99497-F1-model_v4.pdb has 189 a.a.; pLDDT min/med/max: 67.04 98.81 98.97', 'File HSapiens/AF-P00352-F1-model_v4.pdb has 501 a.a.; pLDDT min/med/max: 28.66 98.82 98.98', 'File HSapiens/AF-P21549-F1-model_v4.pdb has 392 a.a.; pLDDT min/med/max: 58.71 98.83 98.98']

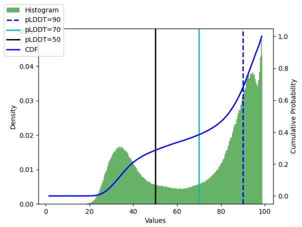

pLDDT all

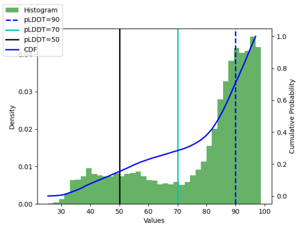

pLDDT median

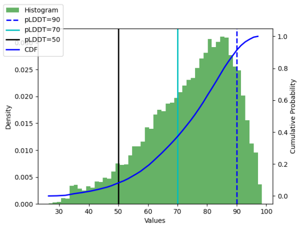

pLDDT mean

Figure 2: **HSapiens: pLDDT statistics**

	50	70	90
HSapiens/all	0.284	0.382	0.666
HSapiens/median	0.139	0.274	0.630
HSapiens/mean	0.071	0.344	0.877

Table 3: **HSapiens: CDF pLDDT**

1.2.2 PAeruginosa

As reported in Figure 3 and Table 4:

- Worst cases, top 3: ['File PAeruginosa/AF-Q9I728-F1-model_v4.pdb has 198 a.a.; pLDDT min/med/max: 20.78 25.56 47.94', 'File PAeruginosa/AF-Q9I2K8-F1-model_v4.pdb has 327 a.a.; pLDDT min/med/max: 20.37 26.57 89.72', 'File PAeruginosa/AF-Q9I665-F1-model_v4.pdb has 224 a.a.; pLDDT min/med/max: 21.50 29.81 73.31']

- Median cases, 3 of them: ['File PAeruginosa/AF-Q9HTB7-F1-model_v4.pdb has 479 a.a.; pLDDT min/med/max: 47.61 95.54 98.73', 'File PAeruginosa/AF-Q9HVR7-F1-model_v4.pdb has 303 a.a.; pLDDT min/med/max: 39.46 95.54 98.50', 'File PAeruginosa/AF-Q9HXS6-F1-model_v4.pdb has 267 a.a.; pLDDT min/med/max: 37.32 95.54 98.81']

- Top cases, top 3: ['File PAeruginosa/AF-Q9HTP2-F1-model_v4.pdb has 497 a.a.; pLDDT min/med/max: 54.80 98.85 98.98', 'File PAeruginosa/AF-Q9HWU0-F1-model_v4.pdb has 461 a.a.; pLDDT min/med/max: 42.05 98.85 98.98', 'File PAeruginosa/AF-Q9I121-F1-model_v4.pdb has 159 a.a.; pLDDT min/med/max: 90.82 98.86 98.96']

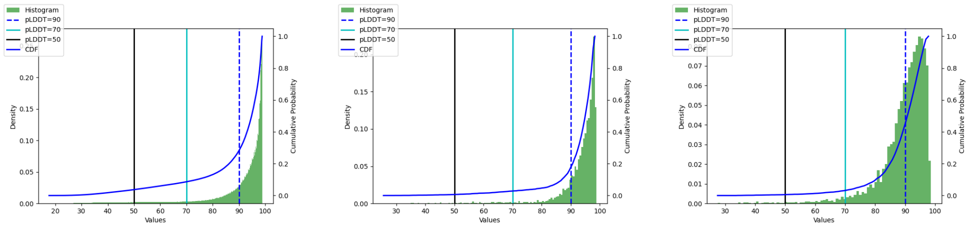

pLDDT all   pLDDT median   pLDDT mean

Figure 3: **PAeruginosa: pLDDT statistics**

	50	70	90
PAeruginosa/all	0.036	0.086	0.278
PAeruginosa/median	0.006	0.028	0.156
PAeruginosa/mean	0.005	0.030	0.396

Table 4: **PAeruginosa: CDF pLDDT**

1.2.3 PFalciparum

As reported in Figure 4 and Table 5:

- Worst cases, top 3: ['File PFalciparum/AF-Q8ILT4-F1-model_v4.pdb has 2280 a.a.; pLDDT min/med/max: 15.91 23.66 96.49', 'File PFalciparum/AF-C0H4Q1-F1-model_v4.pdb has 2580 a.a.; pLDDT min/med/max: 17.34 24.41 96.81', 'File PFalciparum/AF-C6KT74-F1-model_v4.pdb has 2404 a.a.; pLDDT min/med/max: 16.33 24.84 98.69']

- Median cases, 3 of them: ['File PFalciparum/AF-Q8IFL1-F1-model_v4.pdb has 231 a.a.; pLDDT min/med/max: 33.35 76.56 93.89', 'File PFalciparum/AF-Q8I419-F1-model_v4.pdb has 366 a.a.; pLDDT min/med/max: 27.79 76.59 97.05', 'File PFalciparum/AF-Q8IIM7-F1-model_v4.pdb has 698 a.a.; pLDDT min/med/max: 22.18 76.60 95.19']

- Top cases, top 3: ['File PFalciparum/AF-Q8IKK7-F1-model_v4.pdb has 337 a.a.; pLDDT min/med/max: 69.22 98.66 98.94', 'File PFalciparum/AF-Q8IAY6-F1-model_v4.pdb has 198 a.a.; pLDDT min/med/max: 66.94 98.69 98.94', 'File PFalciparum/AF-Q8I3X4-F1-model_v4.pdb has 245 a.a.; pLDDT min/med/max: 49.70 98.76 98.97']

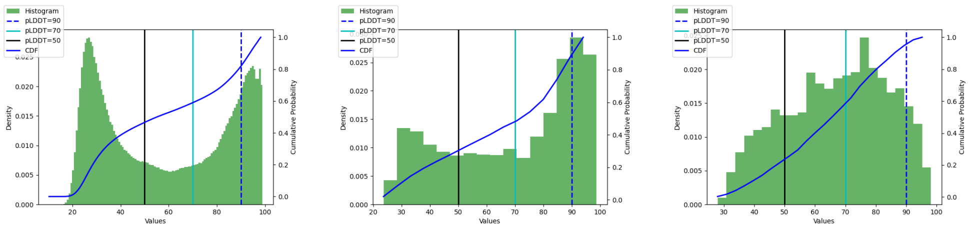

pLDDT all pLDDT median pLDDT mean

Figure 4: **PFalciparum: pLDDT statistics**

	50	70	90
PFalciparum/all	0.460	0.584	0.804
PFalciparum/median	0.261	0.441	0.757
PFalciparum/mean	0.197	0.529	0.915

Table 5: **PFalciparum: CDF pLDDT**
